# Preparation and Properties of PETG Filament Modified with a Metallic Additive

**DOI:** 10.3390/ma18061203

**Published:** 2025-03-07

**Authors:** Piotr Zmuda Trzebiatowski, Tomasz Królikowski, Agnieszka Ubowska, Katarzyna Wilpiszewska

**Affiliations:** 1 Faculty of Mechanical Engineering, Koszalin University of Technology, ul. Śniadeckich 2, 75-453 Koszalin, Poland; piotr.zmuda@tu.koszalin.pl (P.Z.T.); tomasz.krolikowski@tu.koszalin.pl (T.K.); 2 Faculty of Maritime Technology and Transport, West Pomeranian University of Technology in Szczecin, Piastów 41, 71-065 Szczecin, Poland; agnieszka.ubowska@zut.edu.pl; 3 Faculty of Chemical Technology and Engineering, West Pomeranian University of Technology in Szczecin, Pułaskiego 10, 70-322 Szczecin, Poland

**Keywords:** PETG, filament, fused deposition modeling, 316L steel

## Abstract

The materials used as filaments for additive techniques should exhibit various properties depending on the application and the requirements. The motivation for this study was the need to obtain a filament exhibiting appropriate aesthetic (metal-like) and mechanical properties. Glycol-modified poly(ethylene terephthalate) copolymer (PETG) and micrometric steel powder were used for composite preparation. Subsequently, the obtained material was used as a filament for 3D printing, i.e., by fused deposition modeling (FDM) technique. The physicochemical properties of the obtained filaments were determined, such as morphology (roughness), moisture sorption ability, thermal properties, and mechanical performance (tensile and compressive strength). Importantly, the metal filler did not modify the thermal properties of the polyester matrix, indicating that the filament containing steel microfiller could be processed using the same parameters as for neat PETG. The thermal stability was slightly enhanced after steel powder addition (for 13 wt.% content, the temperature of 75% weight loss was 466 °C; for comparison, that for the reference sample was 446 °C). The reinforcing effect of steel microfiller was noted based on mechanical performance measurements. The steel particles acted as a stiffening agent; the highest maximal tensile strength was observed for the composite with 3 wt.% steel powder content (ca. 68 MPa). Further increasing the microfiller load resulted in a slight decrease in the value of this parameter. A different trend was reported considering the compressive strength, i.e., the value of this parameter increased with steel content. Based on the obtained results, the new PETG composites could be applied as structural materials.

## 1. Introduction

In recent years, 3D printing, especially the fused deposition modeling (FDM) technique [1,2,3], has gained importance in engineering, medicine, and construction applications. The choice of material is one of the most significant steps in additive manufacturing. The technique uses thermoplastic polymer materials in the form of a string (filament). Typically, filaments made of poly(lactic acid), acrylonitrile-butadiene-styrene copolymer (ABS), glycol-modified poly(ethylene terephthalate), polystyrene, polycarbonate, and polyamide, but also blends of the above materials [2,4,5]. Each filament has its own set of properties and printing parameters, as well as benefits and limitations. The most important parameters characterizing materials used in additive techniques are their thermal properties and mechanical strength. From the customers’ point of view, visual considerations are also important; hence, materials doped with wood flour, mineral powders, carbon, glass, aramid, and natural cellulose fibers are commonly used. Importantly, applied additives could be obtained from the waste stream of other processes.

The presence of additives significantly affects the physicochemical properties of the obtained material, e.g., adding carbon or glass fibers increases the tensile strength, while the addition of powders, although it negatively affects this parameter, may increase hardness [6]. They play an important role in cost savings. Additives can also improve electrical conductivity [7] and thermal and optical properties [8]. Additionally, introducing additives could affect the shrinkage reduction and the modified crystallization characteristics and as a result change the mechanical properties of obtained materials [9]. Generally, the presence of metallic filler in a polymer matrix could improve the dimensional stability and the thermal and mechanical properties of the material, but some specific purposes could also be found, such as anti-fouling and corrosion-resistant additives [8]. Moreover, polymer composites filled with conductive metal particles exhibit enhanced thermal and electrical conductivity [10]. And last but not least is aesthetics—the product has a metallic gloss.

PET–metal composites can be prepared using various approaches. Metal (e.g., steel, aluminum) can be in the form of a mesh [11], fibers [12], powder [13], or swarf [14]. Generally, polymer–metal hybrid composites exhibit better mechanical performance when compared to neat PET; however, the filler content is important as it determines the usefulness of the composite.

Recently, PETG, a glycol-modified poly (ethylene terephthalate) copolymer, gained interest in additive manufacturing. Introducing glycol comonomer into the copolymer structure allows for a reduction in its tendency to cold crystallization, thus improving the printability and flexibility [15]. PETG exhibits notable tensile toughness, transparency, flexibility, high processability, recyclability, and excellent chemical resistance [7,16]. It can be used for food and water packaging [17]. It is used to obtain high-quality prototypes in robotics or the car industry [16] and as biocompatible material in medicine for anatomical models, e.g., bone models, orthosis, prosthesis, and others [18,19,20]. As PETG is completely recyclable, its use is in agreement with the assumptions of circular economy. Indeed, recycled PETG was successfully applied to filament production [7]. Introducing additives in fiber or powder form to PETG allows for extending the applications of the composite. There are many reports on testing the reinforcing effect of powder, e.g., organophilized montmorillonite [21], activated carbon [22], or fibers such as carbon fibers [23,24,25], Kevlar fibers [25], or silk fibers [18], on PETG-based composites. Recently, a new preparation method was reported, by which continuous carbon fiber PETG filament could be obtained [26]. Importantly, the introduction of the modifying additive into the PETG matrix must not significantly hinder the fabrication procedure by FDM [10].

The motivation for this research on PETG–steel composite was the need to obtain a filament exhibiting appropriate aesthetic (metal-like) and mechanical properties. In following research, this material will be used to obtain gears for a mechanical system. In this work, the effect of metal filler, i.e., steel powder, on the physicochemical properties (morphology, moisture sorption ability, thermal, and mechanical performance) of a PETG–steel composite was evaluated. It was hypothesized that introducing steel powder modifies the properties of the PETG matrix and would allow the manufacturing of a material with enhanced mechanical properties. First, the continuous filament was obtained by melt mixing extrusion. The effect of steel powder content (3–13 wt.%) on the properties of the continuous filament was evaluated. In the next step, the samples were obtained via the fused deposition modeling technique, and the mechanical performance of the prepared samples was tested.

## 2. Materials and Methods

Glycol-modified poly(ethylene terephthalate) (PETG) was purchased from ROSA 3D, Hipolitów, Poland. The austenitic 316L stainless steel powder (gray, spherical, with an average particle diameter of 50 µm, apparent density 4 g/cm^3^) was delivered by Oerlikon Metco, Pfäffikon Switzerland.

The PETG filaments with steel powder were prepared by extrusion as follows (scheme in Figure S1): The PETG granulate and 316L powder were dried in an oven at 65 °C to constant mass. Subsequently, the dry premixture of PETG granulate and appropriate steel powder amount (3 up to 13 wt.%) was prepared. The loose premix was fed into the extruder’s hopper via a screw feeder. A single-screw extruder (screw diameter 32 mm, l/d 32, Zamak EHP 32) equipped with 4 heating zones and a 1.75 mm hole die was applied for PETG composite compounding. The extrusion temperature ranged from 225 °C (feed zone temperature) to 220 °C (nozzle temperature). The linear extrusion speed was 25 m/min.

The 316L steel contents in the composite filaments were 3, 5, 8, and 13 wt.% (samples abbreviated as PETG-3, PETG-5, PETG-8, and PETG-13, respectively). The neat PETG without the steel addition was also extruded as a reference sample (PETG-0). The filaments were subsequently used to obtain the specimens for mechanical tests via the FDM process.

The surface morphology and size distribution of 316L steel particles were determined using a scanning laser microscope KEYENCE, Osaka, Japan, model VK-9700. The LSM film roughness (Ra and Rz) was calculated as the average of three profiles with an interval of 20 μm using the VK Analyzer software VK-9700K.

Phase transitions of the PETG composites were investigated with the DSC technique (DSC Q100 TA Instruments, New Castle, DE, USA). Aluminum hermetic pans (ca. 10 mg of the sample) were used, with nitrogen as a cooling agent and a heating rate of 10 °C/min applied. A standard run at the temperature range from −80 to 300 °C was performed.

The assessment of the thermal stability of the filaments was performed using TGA (TA Instruments) under 25 mL/min airflow. The samples were heated from room temperature up to 900 °C at a heating rate of 10 °C/min.

Five samples (35 mm length) were placed in a desiccator to remove moisture (to steady-state) for moisture absorption tests of the obtained filaments. The samples were then weighed and placed in a climate chamber (humidity 55 ± 2%, temperature 25 ± 2 °C). The samples were weighed 3, 5, 7, 24, 48, 72, 168, and 336 h after being placed in the climatic chamber. The results obtained in this way were substituted for the following formula [27]:

A_t_ = [(M_t_ − M_0_)/M_0_] × 100%, (1)

where A_t_—sorption of moisture after time t [%]; M_0_—mass of the dry sample [g]; M_t_—sample mass after t time: 24, 72, 168, and 336 h [g].

PETG–steel microfiller systems were obtained using modified Ender 3 (by Creality, Shenzhen, China, equipped) with the following:

- An 0.8 mm hardened steel nozzle;
- All-metal hot end;
- Dual-gear extruder;
- Heated glass bed;
- A 32-bit motherboard;
- Custom X carriage with separated airflow for hot end radiator.

The other manufacturing parameters were collected in Table 1.

**Figure 1:**
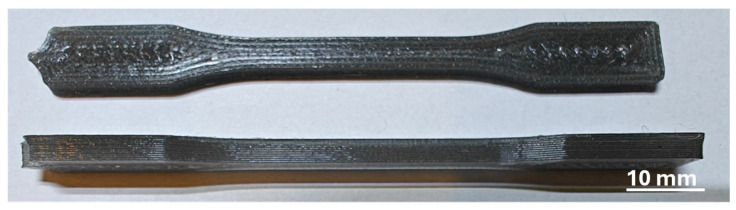
The tensile strength test specimens.

The tensile strength was tested using the Zwick/Roell BT1-FB010TH.D30 testing machine (Ulm, Germany), according to PN-EN ISO 527 standard [28] (the dumbbell sample type 1BA—Figure 1). The gauge length and cross-head speed were 50 mm and 5 mm/min, respectively. The test was performed until the specimen broke or reached an extension of 8% (an extensometer was used). The specimens were obtained from composite filaments via the FDM process. Ten specimens of one material type were tested.

**Figure 2:**
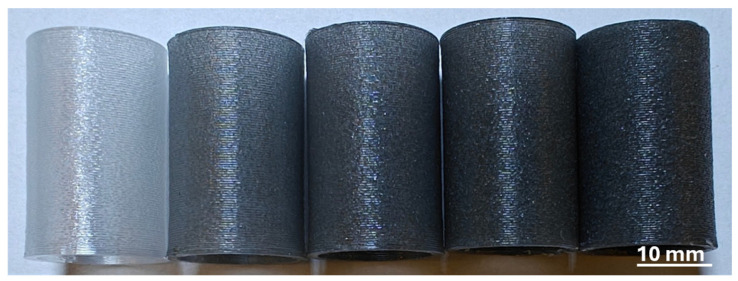
The compressive test specimens.

The compressive strength assessment was performed Inspekt 600, Hegewald and Peschk machine (Nossen, Germany) according to the PN-EN ISO 604 standard [29]. The specimens were obtained using composite filaments via the FDM process. The samples were in the form of a closed circular profile 30 mm high with an external diameter of 19 mm and a wall thickness of 1.2 mm. Due to the specific nature of printing samples in the vase mode, samples were initially taller, and then their upper surfaces were subjected to subtractive processing to ensure the flatness and parallelism of the surfaces touching the plates of the testing device. This also allowed for the standardization of the height of all samples with an accuracy of 0.05 mm. The unusual shape of the sample used, i.e., a thin-walled tube with a longer-than-usual length, allowed for easy optical verification of the samples in terms of manufacturing imperfections that could affect the repeatability of the results. The speed was 1 mm/min until the stress drop indicating permanent failure of the specimen (10%) was reached (machine displacement was used for calculations). At least five samples of each material were tested, and the average impact resistance values were noted. In Figure 2, the samples prepared for the compressive tests are presented.

## 3. Results and Discussion

### 3.1. Morphology

The surface quality of the filaments is an important factor in processing the materials by the fused deposition modeling technique. Information about the microgeometry (surface roughness) allows evaluation of the homogeneity of the composite. Moreover, excessive roughness of the processed material could result in higher friction forces, which, in consequence, could hinder the processing stability and cause more intense wearing of the forming head.

**Figure 3:**
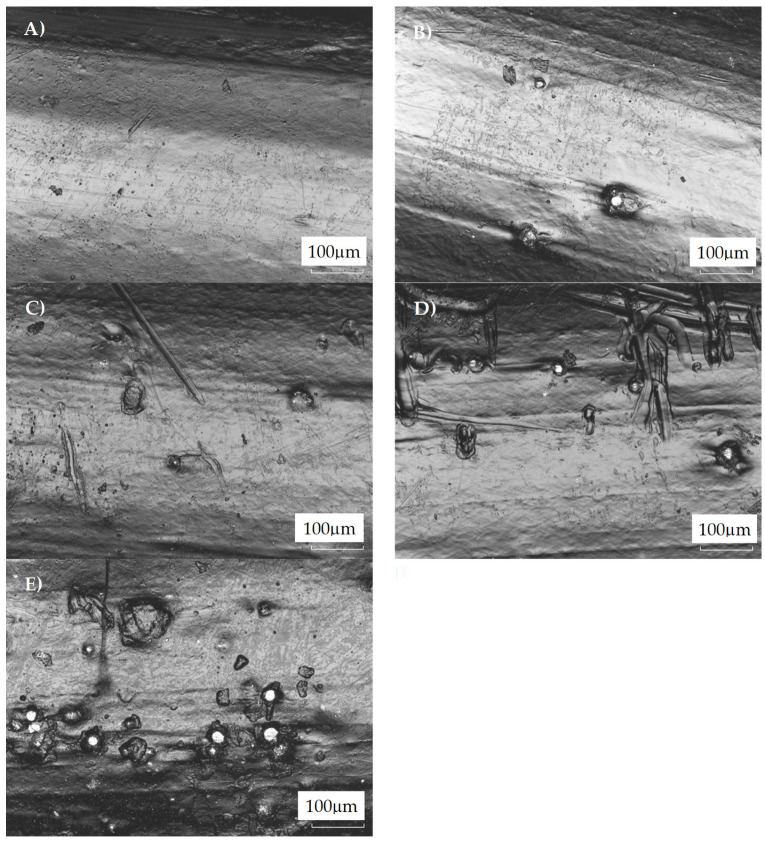
The LSM images of PETG filaments containing (**A**) 0 wt.%, (**B**) 3 wt., (**C**) 5 wt.%, (**D**) 8 wt.%, and (**E**) 13 wt.% steel filler.

In Figure 3, micrographs of the PETG filaments’ surfaces are presented. One could note that the metal particles’ distribution on the filament surface seems nonuniform. The explanation for this could be the different melting temperatures and viscosities of the composite components. At ca. 200 °C, PETG is a very low-viscous fluid, whereas metal powder remains solid. Because of that, the effective mixing is hindered as steel particles tend to sediment. Moreover, it could be noticed that with an increasing amount of steel filler, the surface was more non-uniform. This was the result of the presence of steel particles as well as the scratches caused during the forming of the filaments. The filler particles were noticeable on the filament surface. Moreover, the filler particles do not tend to fall off the surface of the filaments, indicating sufficient adhesion at the PETG–steel interface. The morphology observations strongly correlated with the roughness parameters, i.e., Ra and Rz. The Ra and Rz values increased with steel filler (Table 2). The Ra was determined as the arithmetical mean value, and the Rz values as the sum of the height of the highest profile peak and the depth of the deepest profile valley within an individual measuring distance, respectively [30]. One can conclude that the increased filler load resulted in a more heterogeneous surface of the filaments as a result of the steel particles’ presence noted on the surface of the composite.

The opposite effect was reported for PETG containing carbon fibers [20], i.e., the composite material exhibited significantly reduced roughness. However, the measurements were performed for the specimens after the tensile tests, and “surface smoothness” could be the result of the fibers’ orientation.

### 3.2. Differential Scanning Calorimetry (DSC)

The DSC measurements were performed to determine the effect of steel content on the thermal properties of PETG composites. Neat PETG is an amorphous copolymer, and thus no endo- or exothermic signals for melting or crystallization transitions, respectively, were noted. Thermal characteristics are important, bearing in mind the further processing of the filaments, e.g., shifting the melting point could result in changed flow during extrusion and the processing parameters.

**Figure 4:**
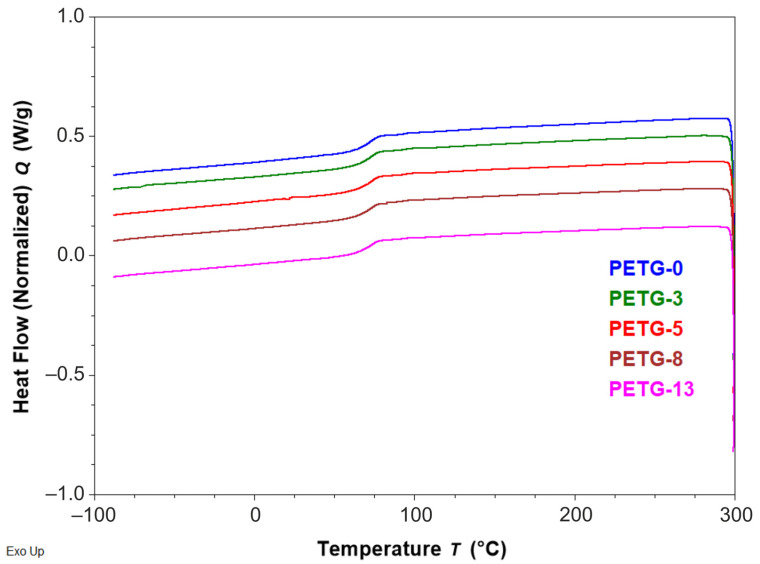
The DSC thermograms of cooling for PETG filaments with various steel filler content.

From the DSC thermograms of the second heating curve (Figure S1) and the cooling curve (Figure 4), the glass transition temperature (Tg ca. 77 °C) was determined (values of these parameters were collected in Table 3), which is in line with the Tg reported by other researchers [26].

Generally, introducing the steel filler in the tested content negligibly affected the thermal parameters of the composite when compared to the reference sample. Moreover, since only one glass transition was observed for all the systems, one can draw the conclusion that no phase separation occurs. Thus, the steel additive did not change the thermal characteristics of the polymer matrix. This is an important feature as it indicates that the filament containing steel microfiller could be processed using the same parameters as neat PETG ones.

As an example, Anis et al. [10] reported the thermal analysis of aluminum-filled PET composites, where the melting and crystallization temperatures shifted towards higher temperatures with increased metal content. As a consequence, the processing parameters should be verified, taking the above into account.

### 3.3. Thermal Stability

Thermogravimetry analysis provides important information about the thermal stability of polymeric materials. It is one of the parameters that could limit the application of the filament. For example, copper powder exhibiting high thermal conductivity and heat capacity, when added to linear low-density polyethylene matrix, caused a substantial decrease in its thermal stability and degradation at lower temperatures [8].

**Figure 5:**
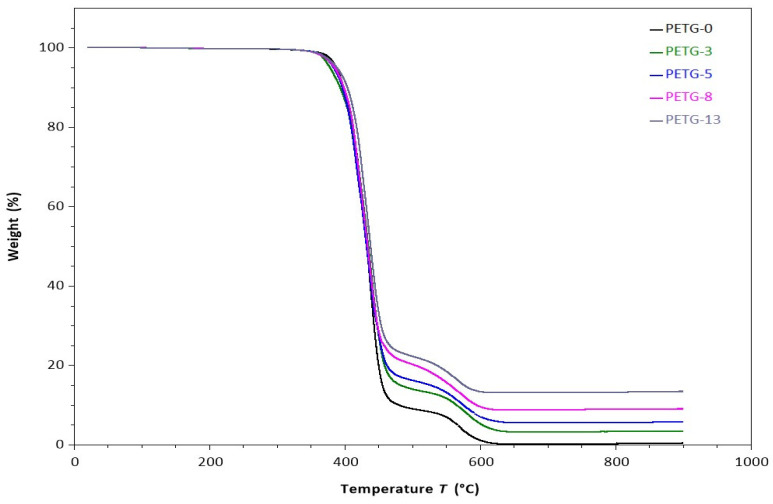
TGA curves of PETG filaments with various steel filler content.

The thermal stability of the obtained systems was studied, and the TG plots are presented in Figure 5. Moreover, the characteristic temperatures of 5, 25, 50, and 75% weight loss are collected in Table 4. All the materials regardless of the microfiller content were thermally stable up to ca. 350 °C. They exhibited similar two-step decomposition patterns when heated to higher temperatures: at 350–470 °C and 500–600 °C. Similar observations for neat PET [31,32], as well as PETG [26], have also been reported. As is known [33], when heated in an air atmosphere, the oxygen molecules attack the flexible and rigid segments, and as a result, ester linkage decomposition occurs. In the second step, the degradation corresponds to breaking the C-C bonds, leading to the formation of volatile substances, e.g., carbon monoxide, carbon dioxide, or low hydrocarbons. Introducing the steel filler into the PETG matrix generally resulted in a slight increase in the thermal stability of obtained filaments: the values of characteristic temperatures of 5, 25, 50, and 75% weight loss shifted toward higher values. This improvement was due to the contribution of the very high thermal resistance of the steel microfiller, which preferably absorbs the heat. A similar effect was reported for high-density polyethylene filled with zinc oxide [34]. The residue content strictly correlated to the steel additive content (Table 4).

### 3.4. Moisture Absorption

**Figure 6:**
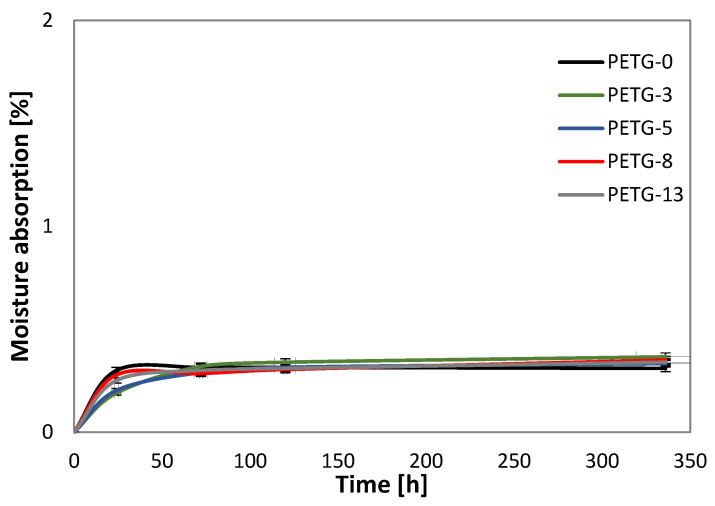
The moisture absorption of PETG filaments with various steel filler contents.

The moisture absorption as a function of storing the filaments at 55% RH is shown in Figure 6. Despite the filler content, all the systems exhibited similar levels of this parameter and did not exceed 0.5%. The highest increase in moisture absorption was noted after 24 h, after which the value of this parameter stabilized. It could be concluded that the steel particles did not affect the moisture absorption characteristics of the matrix, which is an important feature considering the application and processing of the obtained material.

### 3.5. Mechanical Properties

**Figure 7:**
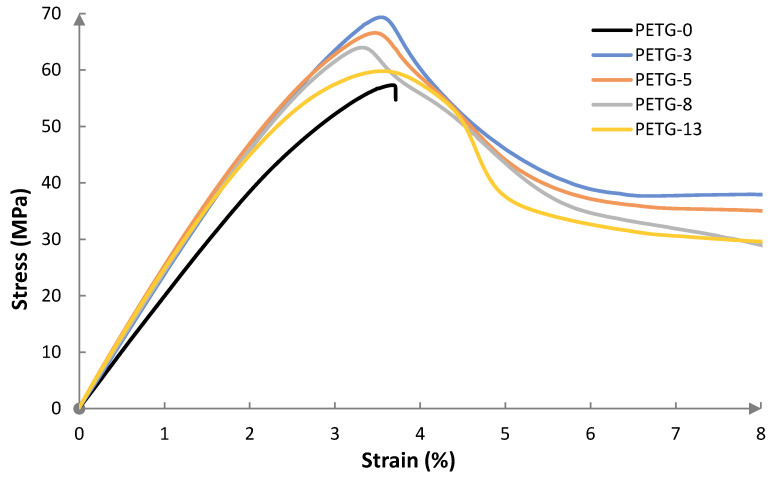
The stress–strain curves of PETG systems with various steel filler contents.

**Figure 8:**
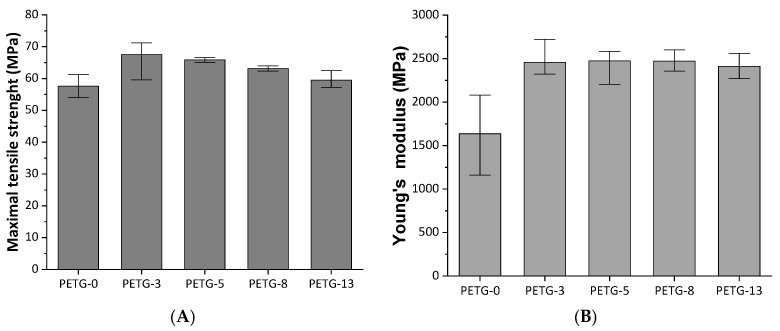
The mechanical strength (**A**) and elastic modulus (**B**) for PETG systems with various steel filler contents.

The mechanical strength is an important parameter for an application viewpoint, especially for constructive materials. The strength properties could be illustrated by the stress–strain curves, shown in Figure 7. The steel content affected the maximal stress of the samples: in Figure 8, the effect of steel filler on the mechanical performance is presented. The measurements were performed for 8% elongation or breaking of the sample.

**Figure 9:**
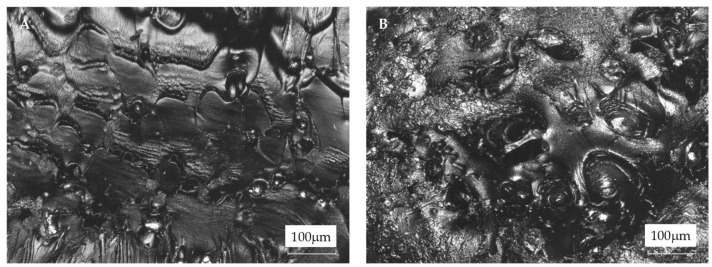
The LSM images of the PETG-13 composite cross-sections (**A**) before and (**B**) after the tensile strength test.

The stress–strain curve for the neat PETG exhibits elastic behavior. The specimen broke after achieving an elongation below 4%, indicating a hard and brittle material. Introducing steel microfiller resulted in modified mechanical characteristics, i.e., the materials remained hard but with reduced brittleness—all the samples exhibited enhanced tensile strength when compared to neat PETG. Interestingly, the highest maximal tensile strength (ca. 20% higher than for neat PETG) was observed for the composite with the lowest steel powder content (3 wt.%). Further increasing the microfiller load resulted in a slight decrease in the value of this parameter. One could conclude that above this given microfiller content, the steel powder tends to aggregate. In Figure 9, images of the cross-sections of the PETG-13 composite before and after tensile strength testing are presented. It can be seen that before the measurement, the steel particles were more or less evenly distributed in the material. However, after the tensile strength measurement, the particles were displaced to the center of the material, in a plane perpendicular to the direction of the tensile force. The particle agglomeration results in a metallic network [14] where the polymer–metal particle interactions are replaced by metal particle–metal particle contact [8]. A similar effect was observed for other thermoplastic matrix systems, such as PE–zinc oxide [34], PE–copper [8], PP–silver powder [35], PVC–aluminum powder [36], and PET–graphene [32], as well as PET–aluminum [26], PETG–activated carbon composites [22], and PETG systems with continuous carbon fibers [26]. 

The common observation is that up to a certain filler content, the tensile strength increases. However, further increasing the filler load results in a drop in mechanical performance. Such overload filler content recalls the plasticizing effect.

When the metallic filler is in the sphere shape, Equation (2) allows the evaluation of the adhesion between the polymer matrix and the metallic filler [34,35]:

(2) $$\frac{\sigma_{c}}{\sigma_{p}}=1-K\Phi_{F}^{2/3}$$

where σ_c_ and σ_p_ are the tensile strength of the composite and the neat matrix, respectively, Φ_F_ is the volume fraction of the filler, and K is the weighting factor depending on the adhesion between the matrix and the filler. Generally, the lower the K value, the better the adhesion [8]. A value of K > 1.2 means that the interphase adhesion is very weak [36]. As an example, the K value for a PP–Ag system was 4.62 [35], and that for LDPE–Cu was 2.03 [8]. Interestingly, the polymers that tend to crystallize exhibited low interaction (adhesion) as a result of low wettability and limited dispersion of the filler in the polymer matrix [35]; thus, the amorphous matrix is preferred for preparing composites with metal particles. The K value for the PETG–steel system calculated as a mean from Equation (2) was below zero, indicating a high degree of interaction between PETG and steel particles [35].

From Figure 8, it can be observed that, when standard deviation is considered, the tensile strength and Young modulus above 3 wt.% of steel powder addition were on a similar level. A similar observation was reported in a case of PET composites containing aluminum powder [10]. This indicates that for the given composite system, some effective filler content level could be noted.

**Figure 10:**
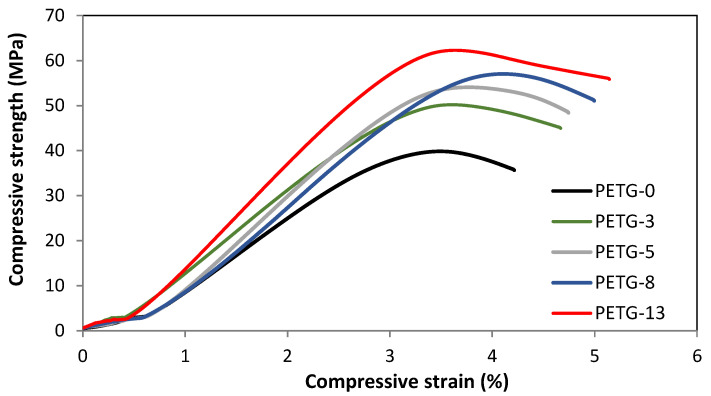
The compressive curves for PETG systems with various steel filler contents.

**Figure 11:**
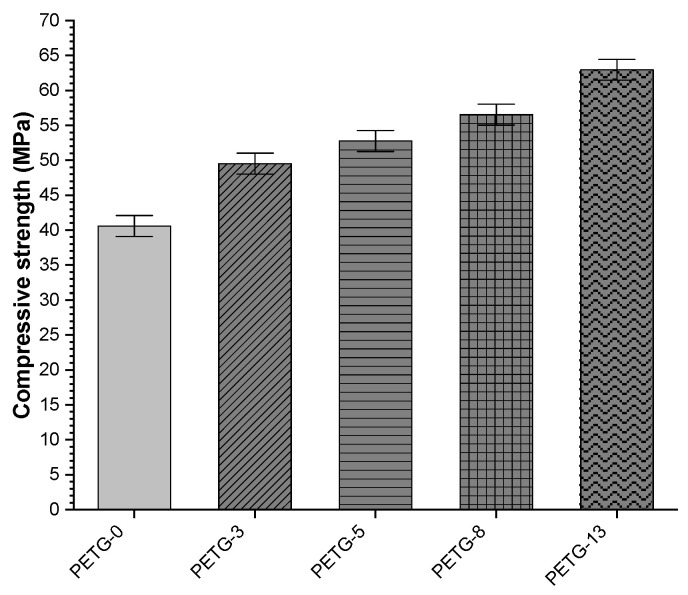
Compressive strength of PETG systems with various steel filler contents.

Compressive properties are essential considering structural materials. In Figure 10, the load–displacement curves are collected for the tested systems. The linear region indicating elastic deformation (i.e., sample returns to the original length when the compressing stress is removed) could be observed for all the materials. However, after reaching the yield point, plastic behavior is observed (i.e., the sample does not return to the original length after removing the stress). The compressive strength increased along with the steel filler content, and the highest value was noted for PETG-13, i.e., of ca. 22 MPa higher when compared to the neat PETG (ca. 62% increase), as shown in Figure 11. Thus, good matrix–steel adhesion and, in consequence, load transfer could be recognized [25].

Similar results, i.e., a compressive strength increase with steel wool powder content, were reported for PET composites prepared via compressing molding [13].

On the other hand, the opposite tendency was described by Valvez et al. [25]. Introducing carbon or Kevlar fibers into the PETG matrix resulted in a significant reduction in compressive strength. Interestingly, using up to 3 wt.% organophilized montmorillonite powder loaded into a PETG matrix allowed the authors obtain a composite with enhanced compressive strength; however, an increased amount of this filler caused a reduction in this parameter.

## 4. Conclusions

The PETG and micrometric steel powder were used in the preparation of composite filaments. Importantly, the metal filler did not modify the thermal properties of the polyester matrix, which suggests that the filament containing steel microfiller could be processed using the same parameters as for neat PETG. The thermal stability was slightly enhanced after the addition of steel powder. The reinforcing effect of steel microfiller was noted based on mechanical performance measurements. The highest maximal tensile strength, 67.5 MPa, was observed for the composite with 3 wt.% steel powder content (i.e., ca. 10 MPa higher than for the reference). Further increasing the microfiller load resulted in a slight decrease in the value of this parameter. A different trend was reported considering the compressive strength, i.e., the value of this parameter increased with steel content. Thus, the steel powder acted as a toughening agent. Based on the obtained results, the new PETG composites could be applied as structural materials. Moreover, the composites containing low conductive filler content could be potentially considered for electrostatic-charge-dissipating applications [10].

Importantly, the obtained PETG composites with steel powder could be processed using regular consumer 3D printers and do not require modified printing parameters (e.g., increased temperature). However, due to the metal powder (friction), prolonged use may cause excessive wear on the nozzle and extruder gears, as well as higher engine and drive load. Thus, it is recommended to use a hardened steel or ruby-tipped nozzle with a diameter of at least 0.6 mm—such nozzles are typically applied for FDM processing of carbon or glass fiber-reinforced filaments.

The obtained PETG–steel filament exhibits metal-like aesthetics and appropriate mechanic properties for the small components of mechanical systems. Thus, in the next step, the prepared materials will be used for the preparation of gears for special purposes. However, to verify this assumption, additional experiments are required.

## Figures and Tables

**Table 1:** The samples; manufacturing parameters for mechanical tests.

Parameter	Value	Additional Comment
Nozzle diameter	0.8 mm	Line width for tensile test 0.88 mm
		Line width for compressive test 1.2 mm
Nozzle temperature	255 °C	
Printing speed	60 mm/s	30 mm/s for outer wall
Temperature of surface bed	80 °C	
Perimeters	2	1 perimeter for compressive test in spiral
		(vase) mode
Layer height	0.32 mm	
Infill	100%	For tensile tests only

**Table 2:** The roughness parameters of the PETG filaments with various filler content.

Filament	Ra (μm)	Rz (μm)
PETG-0	0.562	4.721
PETG-3	2.252	12.091
PETG-5	5.582	53.886
PETG-8	7.360	92.732
PETG-13	9.277	115.301

**Table 3:** The glass transition temperature, crystallization temperature, and crystallization enthalpy for the PETG filaments with various filler content.

Filament	Tg (°C) Second Heating	Tg (°C) Cooling
PETG-0	76.25	77.58
PETG-3	76.47	77.53
PETG-5	76.81	77.46
PETG-8	76.80	76.88
PETG-13	76.86	77.06

**Table 4:** TGA temperatures of 5%, 25%, 50%, and 75% weight loss, and mass residue.

Material	T_5%_ [°C]	T_25%_ [°C]	T_50%_ [°C]	T_75%_ [°C]	Residue [wt.%]
PETG-0	380	416	433	446	0.1
PETG-3	378	416	433	452	3.2
PETG-5	382	413	432	454	5.3
PETG-8	384	416	434	458	8.8
PETG-13	385	421	437	466	13.2

## Data Availability

The original contributions presented in this study are included in the article and Supplementary Materials. Further inquiries can be directed to the corresponding author.

## Supplementary Materials

The following supporting information can be downloaded at: https://www.mdpi.com/article/10.3390/ma18061203/s1, Figure S1: The scheme of preparing PETG/steel composite filaments and the samples for mechanical measurements; Figure S2: The DSC thermograms of 2nd heating for PETG filaments with various steel filler content; Figure S3: The DTG thermograms of PETG filaments with various steel filler content; Figure S4: The images of the PETG-0 and PETG-13 composite before, and after the tensile strength test.
