# Supplementary material for: Preparation and Properties of PETG Filament Modified with a Metallic Additive
Source: Materials (Basel). 2025 Mar 7;18(6):1203. doi: 10.3390/ma18061203 (PMC11943503; doi:10.3390/ma18061203)
Supplement: Supplementary file 1 [file materials-18-01203-s001.zip › materials-3458191-supplementary.pdf]

Supplementary

Preparation and Properties of PETG Filament Modified with a Metallic Additive

Piotr Zmuda Trzebiatowski [1], Tomasz Królikowski [1], Agnieszka Ubowska [2] and Katarzyna Wilpiszewska [3,*]

[1] Faculty of Mechanical Engineering, Koszalin University of Technology, ul. Śniadeckich 2, 75-453 Koszalin, Poland; piotr.zmuda@tu.koszalin.pl (P.Z.T.); tomasz.krolikowski@tu.koszalin.pl (T.K.)
[2] Faculty of Maritime Technology and Transport, West Pomeranian University of Technology in Szczecin, Piastów 41, 71-065 Szczecin, Poland; agnieszka.ubowska@zut.edu.pl
[3] Faculty of Chemical Technology and Engineering, West Pomeranian University of Technology in Szczecin, Pułaskiego 10, 70-322 Szczecin, Poland
* Correspondence: kwilpi@zut.edu.pl; Tel.: +48-91-449-43-73

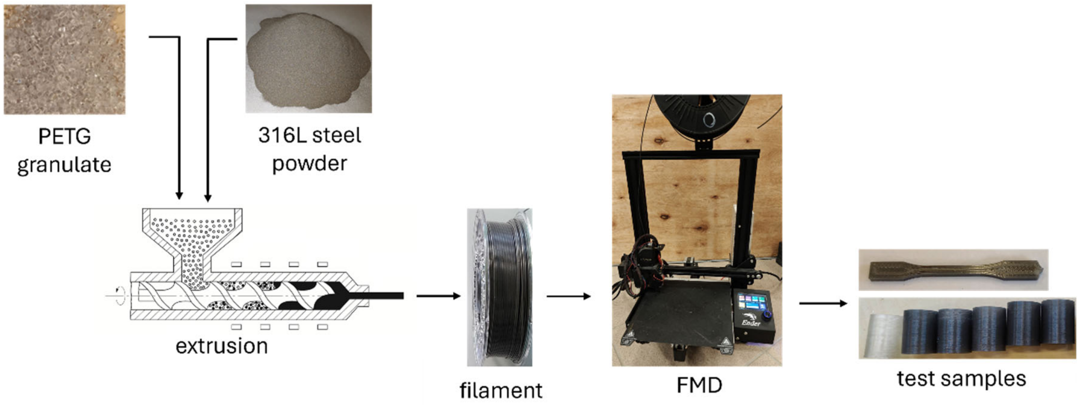

Figure S1. The scheme of preparing PETG/steel composite filaments and the samples for mechanical measurements

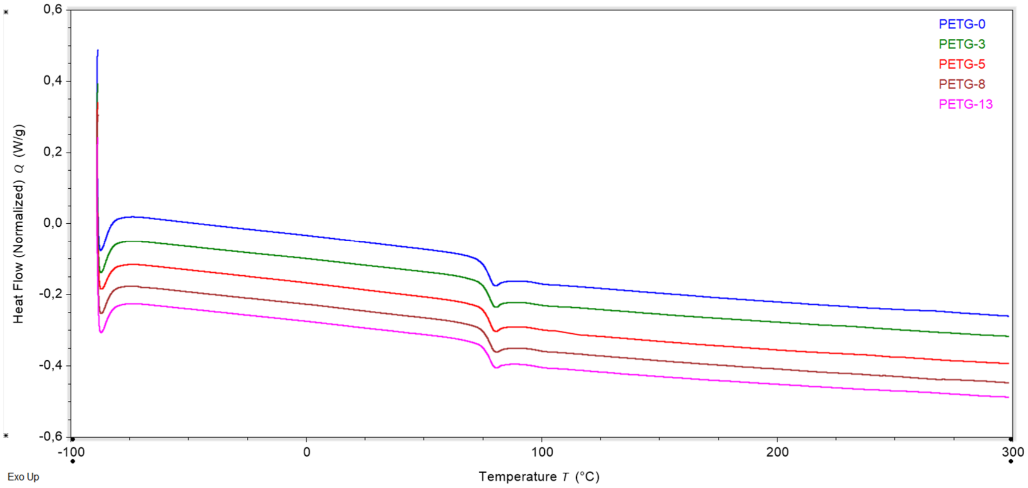

Figure S2. The DSC thermograms of 2nd heating for PETG filaments with various steel filler content

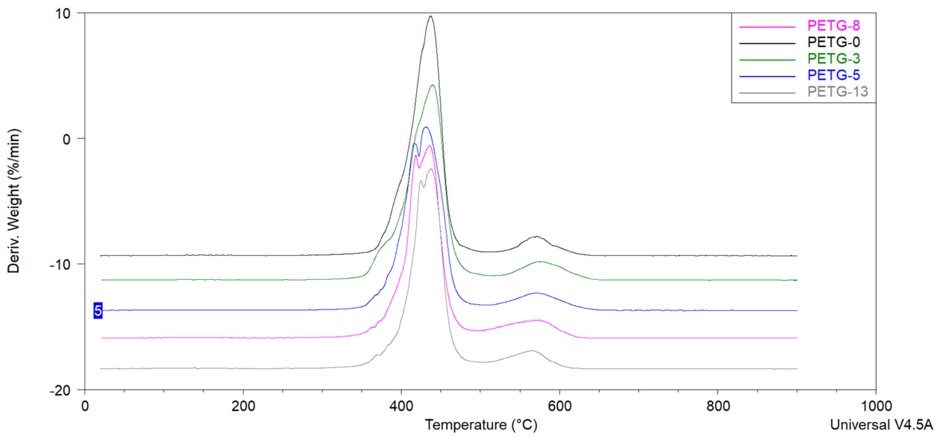

Figure S3. The DTG thermograms of PETG filaments with various steel filler content

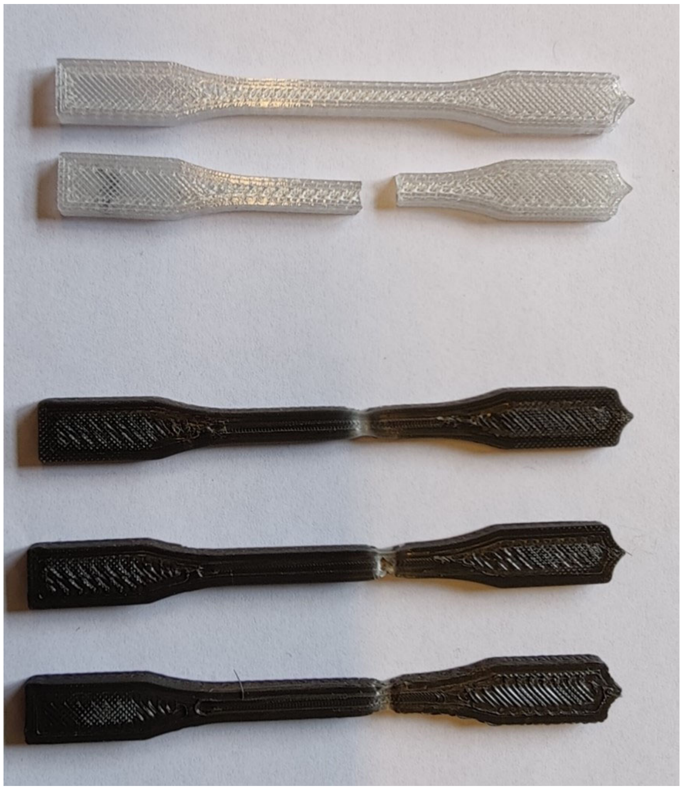

Figure S4. The images of the PETG-0 and PETG-13 composite before, and after the tensile strength test
